# Burns

**Published:** 1854-04

**Authors:** 


*For Burns.*—Use linseed oil and limewater equal parts, shake briskly and saturate a linen or muslin cloth and cover the burnt surface. It is better to mix the ingredients, when required.

